# Case report: Gene mutation analysis and skin imaging of isolated café-au-lait macules

**DOI:** 10.3389/fgene.2023.1126555

**Published:** 2023-03-21

**Authors:** Zhenyu Zhong, Tianhui Yang, Siqi Liu, Shan Wang, Shan Zhou, Shuli Du, Liyun Zheng, Xiuli Wang, Hui Wang, Yifan Wang, Min Gao

**Affiliations:** ^1^ Department of Dermatology, First Affiliated Hospital of Anhui Medical University, Hefei, China; ^2^ Institute of Dermatology, Anhui Medical University, Hefei, China; ^3^ Key Laboratory of Dermatology, Ministry of Education, Anhui Medical University, Hefei, China; ^4^ Anhui Provincial Institute of Translational Medicine, Hefei, China; ^5^ Inflammation and Immune-Mediated Diseases Laboratory of Anhui Province, Hefei, Anhui, China

**Keywords:** isolated café-au-lait macules(CALMs), NF1, dermoscopy, reflectance confocal microscopy, mutation, genetic testing

## Abstract

**Background:** Café-au-lait macules (CALMs) are common birthmarks associated with several genetic syndromes, such as neurofibromatosis type 1 (NF1). Isolated CALMs are defined as multiple café-au-lait macules in patients without any other sign of NF1. Typical CALMs can have predictive significance for NF1, and non-invasive techniques can provide more accurate results for judging whether café-au-lait spots are typical.

**Objectives:** The study aimed to investigate gene mutations in six Chinese Han pedigrees of isolated CALMs and summarize the characteristics of CALMs under dermoscopy and reflectance confocal microscopy (RCM).

**Methods:** In this study, we used Sanger sequencing to test for genetic mutations in six families and whole exome sequencing (WES) in two families. We used dermoscopy and RCM to describe the imaging characteristics of CALMs.

**Results:** In this study, we tested six families for genetic mutations, and two mutations were identified as novel mutations. The first family identified [NC_000017.11(NM_001042492.2):c.7355G>A]. The second family identified [NC_000017.11(NM_001042492.2):c.2739_2740del]. According to genotype-phenotype correlation analyses, proband with frameshift mutation tended to have a larger number of CALMs and a higher rate of having atypical CALMs. Dermoscopy showed uniform and consistent tan-pigmented network patches with poorly defined margins with a lighter color around the hair follicles. Under RCM, the appearance of NF1 comprised the increased pigment granules in the basal layer and significantly increased refraction.

**Conclusion:** A new heterozygous mutation and a new frameshift mutation of NF1 were reported. This article can assist in summarizing the properties of dermoscopy and RCM with CALMs.

## 1 Introduction

Café-au-lait macules (CALMs), also referred to as café-au-lait spots (CALs), are common birthmarks that can be present at birth and dynamically increase in number and range over time. At least one CALM may occur in as many as 2%–3% of healthy newborns (Alper et al., 1979; Alper and Holmes, 1983) and in about one-third of all school-aged children (Whitehouse, 1966; Burwell et al., 1982; Sigg et al., 1990; McLean and Gallagher, 1995; Rivers et al., 1995).

However, having six or more typical CALMs is less common and strongly suggests the diagnosis of neurofibromatosis type 1 (NF1). Isolated CALMs are defined as multiple café-au-lait macules in patients without any other sign of NF1 (Bernier et al., 2016).

NF1 (OMIM 162200) is an autosomal dominant, multisystem disease with an incidence of 1:2,500–3,000 worldwide (Williams et al., 2009). Since 1000 AD, there have been reports of individuals with neurofibromatosis. In 1881, von Recklinghausen coined the name “neurofibroma.” The condition was classified into two types, NF1 and neurofibromatosis type 2(NF2) in the late 1900’s. The National Institutes of Health Consensus Development Conference assembled diagnostic criteria for NF1 that remain utilized in 1988 (Wilson et al., 2021). NF1 is caused by inactivating mutations in *NF1* gene, a tumor suppressor gene mapping to 17q11.2 and encoding neurofibromin. The characteristics of NF1 generally include CALMs, skinfold freckling, Lisch nodules, and neurofibromas.

The diagnostic criteria for NF1 are met in an individual who does not have a parent diagnosed with NF1 if two or more of the following are present:1. ≥6 CALMs over 5 mm in prepubertal and over 15 mm in greatest diameter in postpubertal individuals 2. Freckling in the axillary or inguinal regions 3. Two or more neurofibromas of any type or one plexiform neurofibroma 4. Optic pathway glioma 5. ≥2 iris Lisch nodules identified by slit-lamp examination or ≥2 choroidal anomalies defined as bright, patchy nodules imaged by optical coherence tomography (OCT)/near-infrared reflectance (NIR) imaging 6. A distinctive osseous lesion such as sphenoid dysplasia, anterolateral bowing of the tibia, or pseudarthrosis of a long bone 7. A heterozygous pathogenic NF1 variant with a variant allele fraction of 50% in apparently normal tissue such as white blood cells. Besides, a child of a parent who meets the diagnostic criteria specified in A merits a diagnosis of NF1 if one or more of the criteria in A are present (Legius et al., 2021).

To our knowledge, this is the first study to combine dermoscopy and reflectance confocal microscopy (RCM) to describe the features of CALMs. We combined these two tools aimed at highlighting the use of non-invasive techniques to distinguish typical from atypical CALMs to determine whether the patients should be subjected to further genetic testing.

## 2 Materials and methods

In this study, six Chinese patients with CALMs and their families were recruited from the First Affiliated Hospital of Anhui Medical University in 2022. This study was approved by the Ethical Review Committee of Anhui Medical University and was conducted according to the Declaration of Helsinki guidelines.

For clinical and genetic investigation, all participants signed informed consent forms. The clinical pictures were taken by a digital camera, and lesions were captured under dermoscopy. We also performed the RCM examination, which acquires consecutive series of confocal images from the epidermis to the papillary dermis. Correspondingly, all dermoscopy and RCM images were analyzed appropriately to divide CALMs into typical and atypical.

Peripheral blood samples were taken from the proband and from their parents. According to the manufacturer’s protocol, genomic DNA was extracted from the peripheral blood lymphocytes with kits and stored at −80°C. We used Sanger sequencing to analyze qualified genomic DNA samples. First, we used Primer Premier5.0 to design primers (Supplementary Data Sheet S1) and dilute primers, then we performed PCR amplification. The PCR amplification reaction is completed after the predenaturation stage, amplification stage, and extension phase. The PCR products were purified and recovered after electrophoresis with agarose gels. Finally, we used 3,730 xl sequencer to perform Sanger sequencing and the results were analyzed by DNA sequencing analysis software. We used whole exome sequencing to analyze qualified genomic DNA samples and we got single nucleotide polymorphism (SNP) annotation results. Then, we screened for candidate mutation sites and used SIFT, PolyPhen2, MutationAssessor, MutationTaster and LRT to speculate about the pathogenicity of the mutation sites.

## 3 Results

### 3.1 Basic information about the probands

**FIGURE 1:**
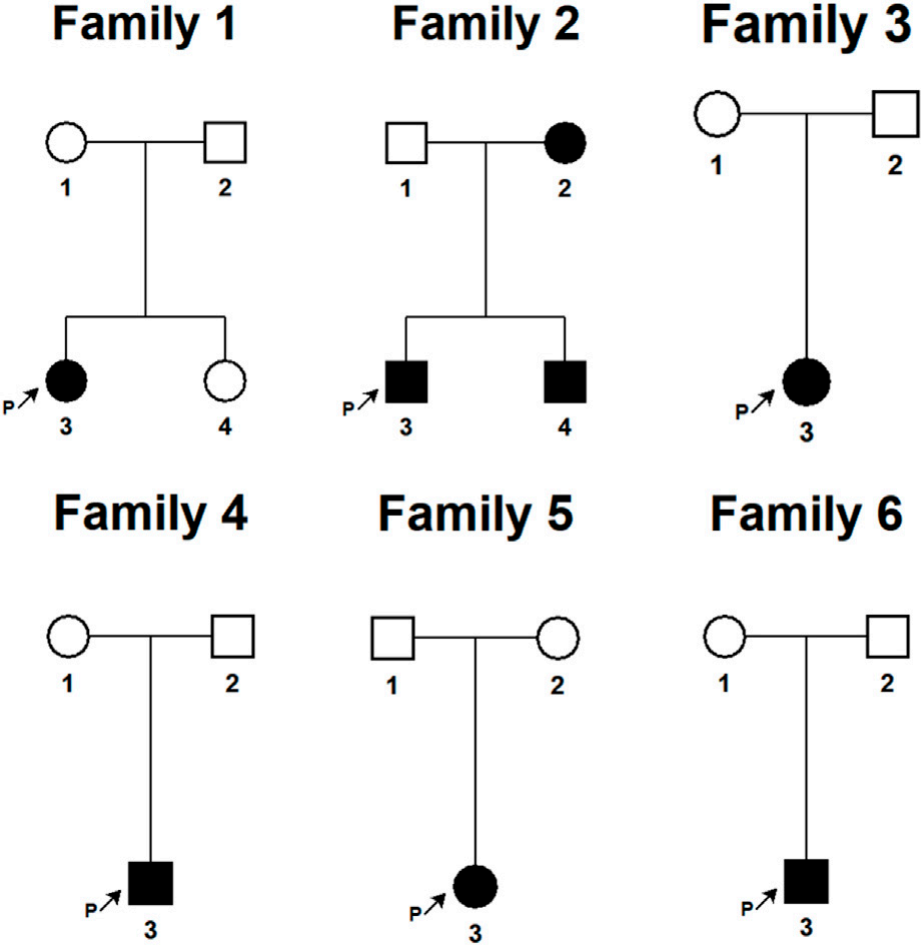
The pedigree Gram of six Chinese Han pedigrees of isolated CALMs. The proband was marked with the arrow. Males were indicated by squares; Females were indicated by circles. Blackened symbols represented patients who had CALMs.

**FIGURE 2:**
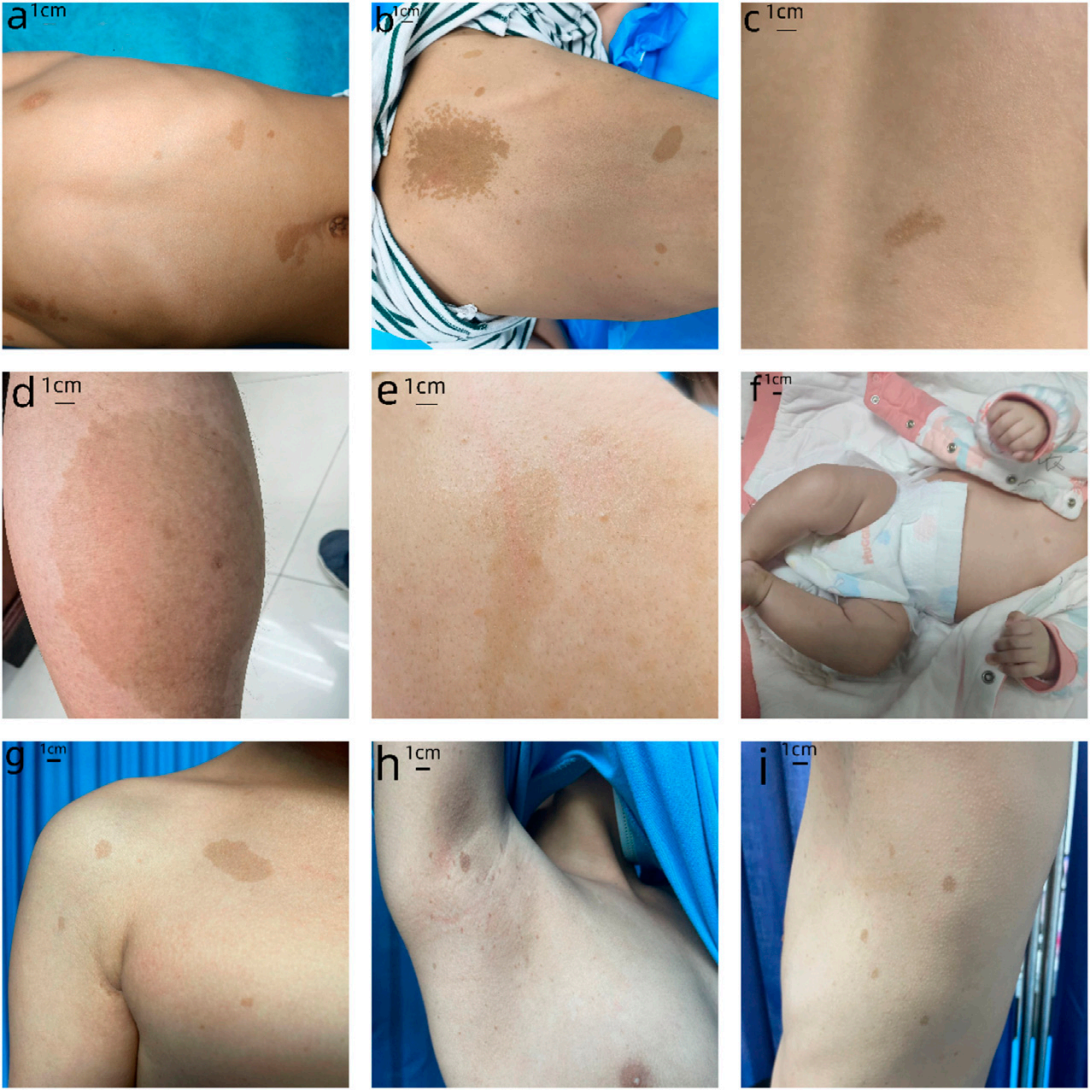
Clinical photographs. **(A)** The first proband: sporadic CALMs were observed in the patient’s abdomen and back since she was born, which spread slowly to the anterior chest and face. **(B)** The second proband: CALMs were observed in the back and hip of the patient since he was born and then gradually spread to the face and neck. **(C)** The brother of the second proband: a pigmented spot with a size of 2 cm was observed on his back. **(D)** The father of the second proband: a pigmented spot with a size of 10 cm was observed on his left calf. **(E)** The mother of the second proband: several scattered pigmentated spots were observed on the back **(F)**The third proband: CALMs were observed on the patient’s chest. **(G)** The fourth proband: CALMs were observed on the patient’s armpit and back. **(H)** The fifth proband: CALMs were observed on the patient’s thorax, back, and legs. **(I)** The sixth proband: CALMs were observed on the patient’s armpit and back.

We have provided the pedigree Gram of the six families (Figure 1). In the first family, the proband, a 7-year-old girl, has several typical CALMs (Figure 2A) and she is the second child of a healthy couple. The patient’s 9-year-old sister, mother, and father had no symptoms at the time of this study. She was otherwise well and had adequate intellectual and physical development. In the second family, the proband, a 7-year-old male has several typical CALMs (Figure 2B) and he is the first child in the family. He has a 5-year-old brother, and his brother also had a pigmented spot on his back (Figure 2C). His father had a large pigmented spot on the left calf (Figure 2D), and his mother had several scattered pigmentated spots on the back (Figure 2E). In the third family, the proband, a female neonate has several typical CALMs (Figure 2F) and she is the first child in the family. The patient’s mother had no symptoms at the time of this study, while her father had CALMs. In the fourth family, the proband, an 8-year-old boy has several typical CALMs (Figure 2G) and he is the only child of his parents. The patient’s parents had no symptoms at the time of this study. In the fifth family, the proband, a 9-year-old girl has several typical CALMs (Figure 2H) and she is the only child of her parents. The patient’s parents had no symptoms at the time of this study as well. In the sixth family, the proband, a 12-year-old boy has several typical CALMs (Figure 2I) and he is the only child of his parents. The patient’s parents also had no symptoms at the time of this study.

### 3.2 Gene mutation analysis

**FIGURE 3:**
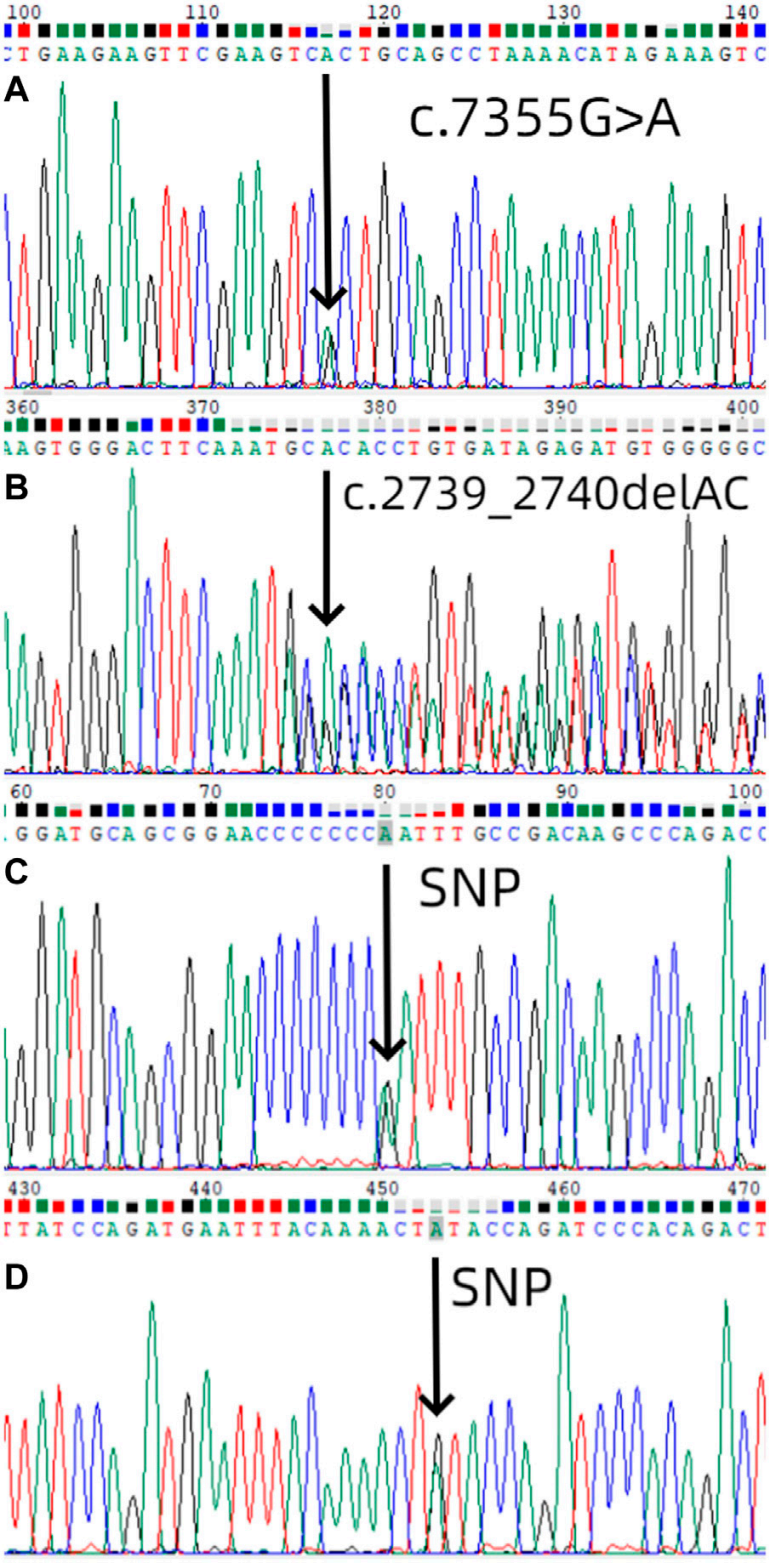
Mutation screening. **(A)** Mutational analysis (next-generation sequencing and direct sequencing for proband) of *NF1* in the first family using DNA sequence of exon 50 of *NF1* gene from the proband, with an arrow showing [NC_000017.11(NM_001042492.2):c.7355G>A]. The sister and the mother of the first proband also identified the same mutation. **(B)** Mutational analysis of *NF1* in the second family using DNA sequence of exon 21 of *NF1* gene from the proband, with an arrow showing [NC_000017.11(NM_001042492.2):c.2739_2740del].**(C)** Mutational analysis of NF1 in the first, fourth, fifth, and sixth families using DNA sequence of exon 18 of NF1 gene from the proband, with an arrow showing [NC_000017.11(NM_001042492.2):c.2034G>A].**(D)** Mutational analysis of NF1 in the second family using DNA sequence of exon 7 of NF1 gene from the proband, with an arrow showing [NC_000017.11(NM_001042492.2):c.702G>A].

We used Sanger sequencing to sequence 9 affected and 11 unaffected members from six families. NF1 gene mutations detected in this study (Table1). In the first family, sequencing of lymphocyte DNA identified [NC_000017.11(NM_001042492.2):c.7355G>A]in the *NF1* gene (Figure 3A). We also performed whole exome sequencing for the first family, and we found the same mutation in the result. All four members in this family received genetic testing and this variant of *NF1* was also identified in her mother and sister but not her father. In the second family, sequencing of lymphocyte DNA identified [NC_000017.11(NM_001042492.2):c.2739_2740del] (Figure 3B). All four members in this family received genetic testing and only the proband identified this mutation. No possible causative mutations in *NF1* were identified in the other four probands and their parents. The proband of the first, fourth, fifth, and sixth families identified [NC_000017.11(NM_001042492.2):c.2034G>A] (Figure 3C). The proband of the second family identified [NC_000017.11(NM_001042492.2):c.702G>A] (Figure 3D).

**TABLE 1:** NF1 gene mutations detected in this study.

Family	Individuals	Exon	Mutation type	Nucleotide mutation	Protein alteration
1	Family1 (1,3,4)	50	Missense	c.7355G>A	p. Arg2452His
	Family1 (3)	18	SNP	c.2034G>A	p. P678P
2	Family2 (3)	21	Frameshift	c.2739_2740delAC	p. I913Mfs*5
	Family2 (3)	7	SNP	c.702G>A	p. L234L
4	Family4 (3)	18	SNP	c.2034G>A	p. P678P
5	Family5 (3)	18	SNP	c.2034G>A	p. P678P
6	Family6 (3)	18	SNP	c.2034G>A	p. P678P

We performed whole exome sequencing on a total of seven people from the first and third families (four in the first family and three in the third family). High-throughput sequencing of hybrid libraries using BGI’s platform yielded an average of 143,344,087 clean reads (14,334,408,742 bps) per sample. Each sample’s clean readings were matched to the sequencing of the human reference genome. We used the results of the GATK comparison pair for SNP detection and annotated the results of the SNP. All samples had an average of 122,655 SNPs, of which 99.46% were discovered in the dbSNP database and 93.69% in the 1000 Genomes Project database. There are 660 SNPs that were just found. Base conversion to base switching is compared in a 2.26:1 ratio (Ti/TV). In the coding region, there are typically 10,812 synonymous mutations, 10,164 missense mutations, 27 SNPs that turn the stop codon into a non-stop codon, 95 SNPs that turn the codon into a stop codon, 33 SNPs that invalidate the start codon, and 2,711 SNPs that alter the shear acceptor or splicing donor in the region surrounding the splicing site. The encoder area SNP’s Ti/TV is 2.99. We analyzed the results of WES and in the first family [NC_000017.11(NM_001042492.2):c.7355G>A]was identified in the proband, her mother and her sister but not her father. (Supplementary Data Sheet S2)In the third family, no likely pathogenic mutations were identified.

Mutations are classified as pathogenic, likely pathogenic, uncertain significance, likely benign, and benign. In the first family, family members all identified [NC_000017.11(NM_001042492.2):c.7355G>A] except his father. We used a variety of software to analyze, the MutationTaster_score is 1, the MutationTaster_pred is D, the MutationAssessor is M, the LRT is D, SIFT score is 0.003 which is less than 0.05, PolyPhen2 is 0.989 which is greater than 0.909. According to ACMG Standards and Guidelines, the evidence of pathogenicity of this mutation is very strong (PVS1), so this mutation is classified as pathogenic. In the second family, only the proband identified [NC_000017.11(NM_001042492.2):c.2739_2740del]. We used the MutationTaster to analyze this mutation and it predicted this mutation as pathogenic and the probability is 1 because this mutation lead to non-sense-mediated mRNA decay (NMD). In the analysis, Phylop is a positive number and PhastCons is 1 which means this mutation occurred in conserved sequences. According to ACMG Standards and Guidelines, the evidence of pathogenicity of this mutation is very strong (PVS1), so this mutation is classified as pathogenic. [NC_000017.11(NM_001042492.2):c.2034G>A]and [NC_000017.11(NM_001042492.2):c.702G>A] were analyzed by SIFT, PolyPhen2 and MutationTaster as Polymorphism (SNP), which were not pathogenic.

According to *NF1* genotype-phenotypic correlation analyses, a higher number of CALMs was observed in patients with truncated mutations compared to patients with missense mutations (Sabbagh et al., 2013). According to our observation, the patient in the second family had more CALMs than the patient in the first family, which complied with the result above. Besides, proband with frameshift mutation tended to have a higher rate of having atypical CALMs.

We summarized the characteristics of CALMs in all affected individuals based on the type of CALMs (typical or atypical), the number of CALMs, and mutations in *NF1* gene (Table 2).

**TABLE 2:** The characteristics of CALMs in all affected individuals.

	Typical or atypical	Number of calms	Mutations in *NF1* gene
The first proband	Typical	About 15	Missense mutations in *NF1* gene
The second proband	Typical and atypical	>50	frameshift mutation of *NF1*
Brother of the second proband	Typical	1	Not found
Father of the second proband	typical	1	Not found
Mother of the second proband	Typical	About 5	Not found
The third proband	Typical	About 10	Not found
The fourth proband	Typical	5	Not found
The fifth proband	Typical and atypical	About 20	Not found
The sixth proband	Typical	About 10	Not found

### 3.3 Skin images

**FIGURE 4:**
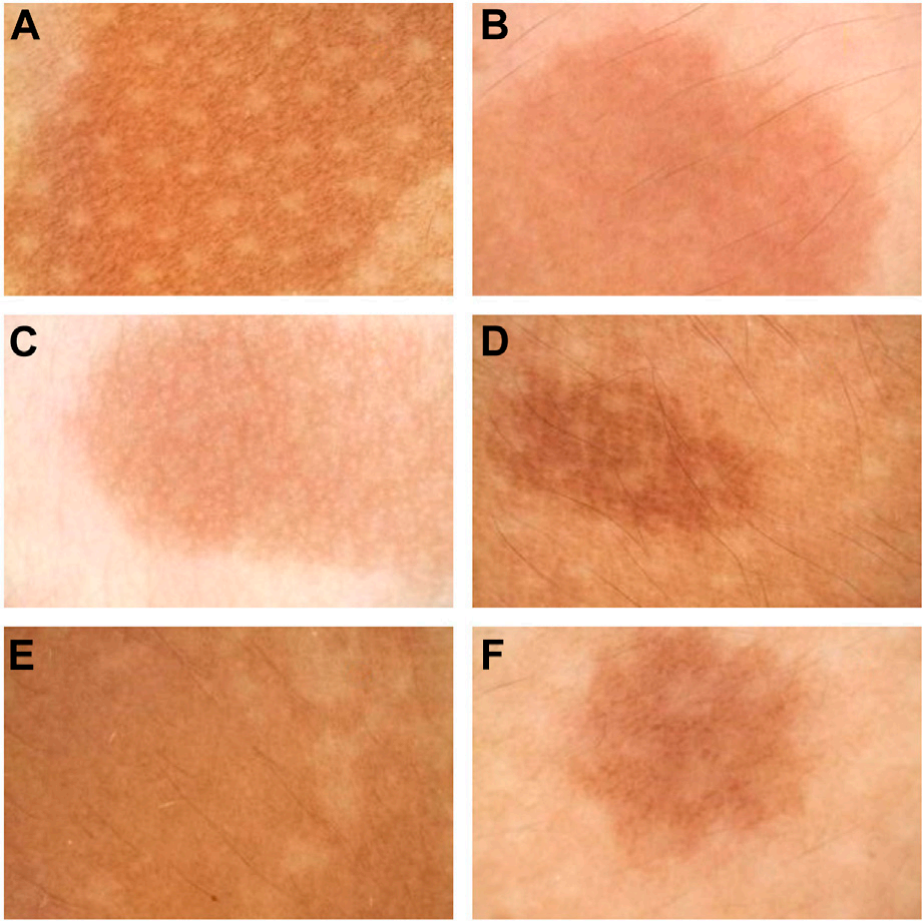
Dermoscopy pictures. **(A)** The dermoscopy picture of the first proband. **(B)** The dermoscopy picture of the second proband. **(C)** The dermoscopy picture of the third proband. **(D)** The dermoscopy picture of the fourth proband. **(E)** The dermoscopy pictures of the fifth proband. **(F)** The dermoscopy pictures of the sixth proband.

The features of CALMs, as seen under dermoscopy, were visible brown pigment patches with clear borders and different sizes (Figure 4).

**FIGURE 5:**
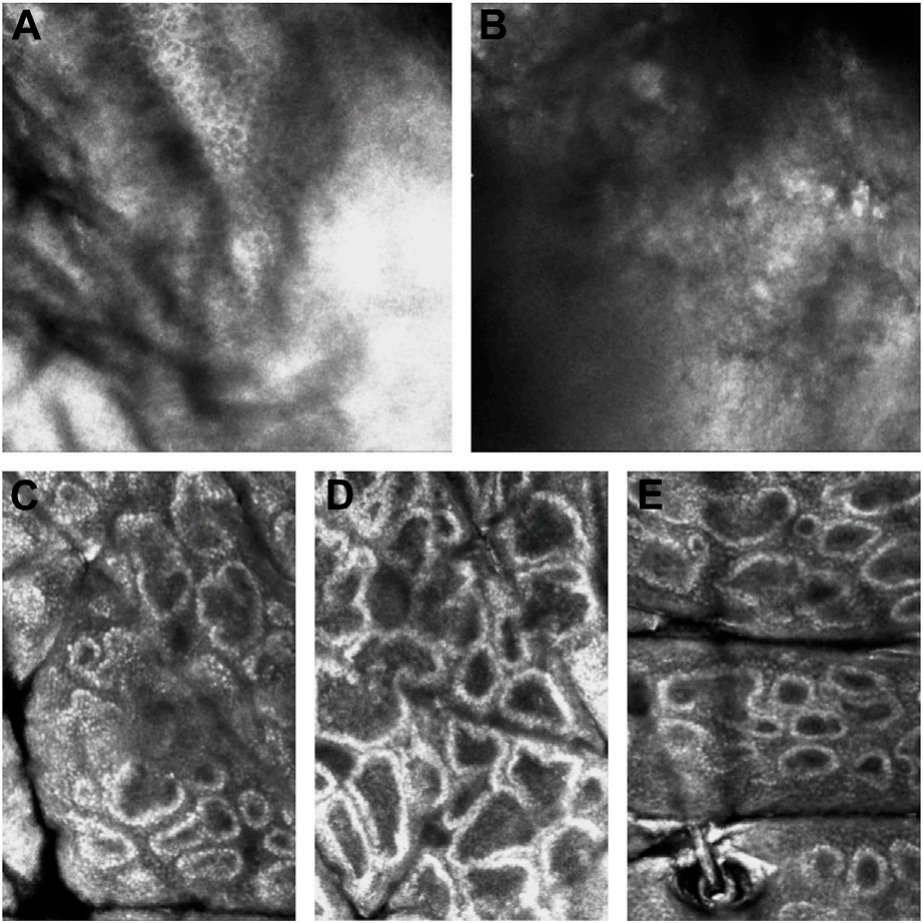
RCM pictures. **(A)** The RCM pictures of the first proband. **(B)** The RCM pictures of the second proband. **(C)** The RCM pictures of the fourth proband. **(D)** The RCM pictures of the fifth proband. **(E)** The RCM pictures of the sixth proband. The third proband did not have the RCM images due to the patient’s parents refusal.

The features of CALMs, as seen under RCM, were increased pigment content in the basal layer in the skin lesion area, many highly refractive particles, and no significant changes in the superficial dermis (Figure 5).

## 4 Discussion

CALMs are flat, well-circumscribed light-to-dark brown macules or patches on the skin; however, the lesions can vary in pigmentation. CALMs can be divided into typical and atypical CAL spots based on number, morphology, and pigmentation (Fois et al., 1993; Nunley et al., 2009; Riccardi, 2009; St John et al., 2016; Albaghdadi et al., 2022). Typical CALMs are described as round and smooth with a size of 5 mm–15 cm, and their pigmentation is even and uniform. Atypical CALMs are described as irregular and jagged with a size of <5 mm or >15 cm, and their pigmentation is uneven and non-homogeneous.

CALMs associated with NF-1 are described as typical (Nunley et al., 2009). Previous studies have also shown that typical CALMs have more predictive value for NF-1 than atypical CALMs (Fois et al., 1993; Nunley et al., 2009; Riccardi, 2009). Identifying typical CALMs can serve as a preliminary basis for genetic testing; however, we need further studies with larger samples to verify this.

The pathogenesis of diseases in which CALMs are seen mainly includes the abnormalities in the Ras/MAPK signaling pathway and KITLG/KIT signaling pathway, which are involved in different diseases that cause CALMs (Table 3) (Zhang et al., 2016).

**TABLE 3:** Different diseases that cause CALMs.

Syndrome	Gene or locus	Location	Protein	Protein function	Clinical features
RAS/MAPK signaling pathway NF1	NF1	17q11.2	Neurofibromin	RasGAP	Cafe-au-lait maculae skinfold freckling, Lisch nodules, neurofibromas
Legius syndrome	*SPRED1*	15q14	SPRED1	SPROUTY-related, EVH1 domain-containing protein 1	Multiple CALs with or without intertriginous freckling macrocephaly
Noonan Syndro me	*PTPN11*	12q24.1	SHP2	Phosphatase, RasGEF, tyrosine kinases,serine-threoninekinases	Characteristic facial features, lentigines short stature, congenital heart defects
	*SOS1*	2p22.1	SOS1		
	*RAF1*				
	*KRAS*				
	*NRAS*				
	*BRAF*				
	*SHOC2*				
	*CBL*				
	*RIT1*				
	*MAP2K1*				
	*SOS2*				
	*LZTR1*				
	*A2ML1*				
	*RRAS*				
	*RASA2*				
Noonan syndrome With lentigines	*PTPN11*	12q24.1	SHP2	Phosphatase	Multiple lentigines plus the features of Noonan syndrome
Mccune-Albright syndrome (mas)	*GNAS1*	GNAS1	cAMP pathway-associated G-protein	α-subunit’s intrinsic GTPase	CALMs, skeletal abnormalities, endocrine disorders
KITLG/KIT signaling pathway					
Piebaldism	*KIT*	4q12	KIT ligand (stem cell factor, steel factor or mast cell growth factor)and its receptor KIT	Ras/mitogen activated protein kinase (MAPK)	Depigmented patches of skin and hair
Familial progressive hyperpigmentation (FPH)	*KITLG*	12q21.32	KIT ligand (stem cell factor, steel factor or mast cell growth factor)and its receptor KIT	Ras/mitogen activated protein kinase (MAPK)	Diffuse, partly blotchy hyperpigmented lesions intermingled with scattered hypopigmentations, and lentigines

In our study, the probands all had only CALMs without other manifestations that suggest other diseases; therefore, we only tested them for genetic mutations in *NF1* gene. If other manifestations develop in these probands, then we will test them for mutations in other genes according to their signs and symptoms, followed by a long follow-up.

A novel missense mutation was identified in the proband in the first family, who had about 15 typical CALMs. A novel frameshift mutation was identified in the proband in the second family, who had over 50 typical CALMs and several atypical CALMs. The literature has shown that probands with a frameshift mutation tend to have a higher rate of having atypical CALMs and a larger number of CALMs than probands with a missense mutation, and our research is consistent with this rule.

In the first family, the proband identified [NC_000017.11(NM_001042492.2):c.7355G>A]and this variant of *NF1* was also identified in her mother and sister but not her father. But as far as we observed, the mother and sister of the proband had no symptoms. As we all know, NF1 is an autosomal dominant disorder. According to the medical genetics, heterozygotes with dominant pathogenic genes show disease only after reaching a certain age because the pathogenic genes are not expressed or are not expressed enough to cause obvious clinical manifestations in early lifetime, which is called delayed dominance. What’s more, some dominant genes of heterozygotes exhibit the associated dominant qualities, but they can be inconspicuous in others, or they do not express the corresponding traits, which is called irregular dominance. We speculate that the phenomenon in the first family is related to these two conditions. The inheritance of gene mutation traits is influenced by the environment and family background, and therefore appears diverse.

We performed Sanger sequencing on all members of the six families, and whole exome sequencing on all members of the first and third families. Only the first two families found possible causative mutations, possible causative mutations were not identified in the other four families. Whole exome sequencing has been increasingly adopted as the primary genetic diagnostic strategy for the past few years due to its high diagnostic yield and cost-effectiveness (Qiu et al., 2022). But WES cannot provide hitherto complete coverage of the coding region of the genome like whole genome sequencing (WGS). According to the literature, about 10% of the mutations detectable by whole exome sequencing (WES) were missed (Meienberg et al., 2016). The NF1 genome is large and has many hundreds of thousands of bases, using Sanger sequencing cannot completely cover these bases. We speculate that this was one of the reasons why the other four family identified no possible causative mutations. Another reason was that the mutations in these four patients might have been in genes other than *NF1*, resulting in other diseases causing CALMs that we have enumerated above. As a result, it is necessary to broaden the scope of genetic testing and test for more genes, such as *SPRED1* and *NF2*.

Dermoscopy is an important non-invasive technique to diagnose cutaneous pigmented diseases, which can magnify lesions to observe the color from the epidermis to the papillary layer. RCM can be used to image skin to a depth of 250 m (superficial dermis) and has been proven to be a critical non-invasive technique for diagnosing melanocytic lesions. The RCM characteristics of CALMs have been summarized in previous articles, but this article is the first to summarize both dermoscopy and RCM characteristics of CALMs.

Nowadays, as clinicians generally observe the number and shape of CALMs macroscopically, we believe that dermoscopy and RCM can be used as a preliminary basis to examine whether CAL spots are typical and decide whether genetic testing is needed. Thus, we sorted out the characteristics of skin imaging of CALMs to help clinicians make the decision. According to the dermoscopy and RCM results of these six patients with CALMs, we could preliminarily conclude the characteristics of CALMs. Under dermoscopy, CALMs usually manifest as brown pigment spots with a clear rim. Under RCM, CALMs usually demonstrated an increased pigment content in the basal layer of the skin lesion area and no obvious changes in the superficial dermis.

## Data Availability

The datasets for this article are not publicly available due to concerns regarding participant/patient anonymity. Requests to access the datasets should be directed to the corresponding author.
